# Regulatory T Cell Stability and Plasticity in Atherosclerosis

**DOI:** 10.3390/cells9122665

**Published:** 2020-12-11

**Authors:** Amal J. Ali, Jeffrey Makings, Klaus Ley

**Affiliations:** 1 Laboratory of Inflammation Biology, La Jolla Institute for Immunology, 9420, Athena Circle Drive, La Jolla, San Diego, CA 92037, USA; aali@lji.org (A.J.A.); jmakings@lji.org (J.M.); 2 Department of Bioengineering, University of California, 9500 Gilman Drive, MC0412, La Jolla, San Diego, CA 92093, USA

**Keywords:** atherosclerosis, Tregs, stability

## Abstract

Regulatory T cells (Tregs) express the lineage-defining transcription factor FoxP3 and play crucial roles in self-tolerance and immune homeostasis. Thymic tTregs are selected based on affinity for self-antigens and are stable under most conditions. Peripheral pTregs differentiate from conventional CD4 T cells under the influence of TGF-β and other cytokines and are less stable. Treg plasticity refers to their ability to inducibly express molecules characteristic of helper CD4 T cell lineages like T-helper (Th)_1_, Th_2_, Th_17_ or follicular helper T cells. Plastic Tregs retain FoxP3 and are thought to be specialized regulators for “their” lineage. Unstable Tregs lose FoxP3 and switch to become exTregs, which acquire pro-inflammatory T-helper cell programs. Atherosclerosis with systemic hyperlipidemia, hypercholesterolemia, inflammatory cytokines, and local hypoxia provides an environment that is likely conducive to Tregs switching to exTregs.

## 1. Introduction

Atherosclerosis is the leading cause of death globally, with about 610,000 deaths in the US annually. Atherosclerosis is a chronic progressive inflammatory disease with an autoimmune component [1]. Two recent clinical trials (CANTOS and COLCOT) show that anti-inflammatory therapies can have beneficial effects for outcomes of atherosclerosis, including myocardial infarction, cardiovascular death, and stroke [2,3]. Inflammation in the atherosclerotic vessel wall is driven by both the adaptive and innate immune responses [4,5,6,7], and regulatory T cells (Tregs) have attracted considerable attention as immune modulators in cardiovascular disease [8]. 

CD4 T cells help with killing and interferon-γ-driven type 1 inflammation (T-helper Th_1_); IL-4-, 5- and 13-driven type 2 inflammation (Th_2_); IL-17-driven inflammation (Th_17_), maturation of germinal centers; isotype switching; and affinity maturation of B cells (follicular helper T_fh_) and other subsets. Tregs are a subset of CD4 T cells that maintain immunological homeostasis by suppressing the functional activity of conventional effector T cells (Tcons). The majority of CD4^+^ Tregs are generated in the thymus (tTregs), and others can be generated from peripheral Tcon cells (pTregs). tTregs arise from CD4^+^CD25^+^ cells upon engagement of their high-affinity, self-reactive T-cell receptors (TCRs) [9]. tTregs are found primarily in lymphoid tissues and blood [10] and can migrate to inflamed non-lymphoid tissues. pTregs, on the other hand, arise when peripheral CD4^+^CD25^−^ Tcons are exposed to IL-2 and TGF-β in vitro or to an unknown cytokine mix in vivo. pTregs are typically found in mucosal tissues and are responsible for damping local inflammation elicited by foreign antigens [11,12,13,14]. Both tTregs and pTregs maintain self-tolerance and suppress the activity of CD4^+^CD25^−^ Tcons via IL-10 and TGF-β production and through cell–cell interactions. Another subset of peripherally induced CD4^+^ Tregs, termed T-helper 3 (Th_3_) [15] cells, suppress the proliferation and cytokine secretion by Th_1_ and Th_2_ cells in a TGF-β-dependent, but not an IL-10-dependent, manner. A fourth subset are Tr1 cells, a cell type that mainly suppresses immune responses by secreting IL-10 and TGF-β [16]. In human Tregs, IL-7R(CD127) expression is negatively correlated with Foxp3 expression; thus, the common phenotype of human Tregs is CD4^+^CD25^hi^Foxp3^+^CD127^low^ [16].

tTregs and pTregs, but not Th3 or Tr1 cells, express the Forkhead box P3 (Foxp3) transcription factor. In mice, their phenotype is CD4^+^CD25^+^Foxp3^+^. Foxp3 is the only Treg-defining transcription factor that has been identified to date. Loss of Foxp3 results in defective Treg development and causes fatal autoimmune diseases in mice and humans [17,18,19]. Nonetheless, the hypothesis that Foxp3 is the sole defining factor for the Treg lineage has been challenged by several studies. On one hand, Foxp3 transduction into CD4^+^CD25^−^ is not sufficient to fully recapitulate the transcriptional landscape of Tregs [20,21]. On the other hand, in mice in which a stop codon was introduced into Foxp3 and no FoxP3 protein was made, Foxp3-deficient T cells still expressed some of the Treg cell signature genes [22]. Unknown transcriptional regulator(s) may be upstream of Foxp3 [23]. Tr1 cells arise from CD4^+^CD25^−/low^ when the immune system is experiencing a chronic inflammatory response in the presence of IL-10 [24]. Although Tr1 cells lack Foxp3, they dampen cytokine secretion and proliferation of naive CD4^+^CD25^−^T cells, including Th_1_ and Th_2_ cells in an IL-10- and TGF-β-dependent manner. 

Given that Foxp3 is considered the master regulator of Tregs and its absence can result in severe autoimmunity, it is reasonable to assume that the long-term lineage stability of Tregs is highly dependent on the stability of Foxp3 expression. Foxp3 imposes a unique transcriptional signature on Tregs by interacting, directly or indirectly, with cell surface proteins, signaling molecules, transcription factors, non-coding RNAs, and epigenetic regulators [22,25,26]. The *Foxp3* locus encodes three evolutionarily conserved non-coding sequences (CNS1–3) that determine the size and stability of the Treg pool [27]. CNS3 is the main inducer of Foxp3 expression in tTregs and pTregs. The maintenance of Foxp3 expression in tTregs is epigenetically regulated by CNS2, also known as Treg-specific demethylated region (TSDR) [27,28,29]. During early stages of thymic Treg development, signaling through IL-2 and other γ-chain cytokines initiates TSDR demethylation and thereby regulates Foxp3 expression [30,31]. Moreover, loss of CNS2 inhibits the heritable expression of Foxp3 when mature Tregs divide under inflammatory conditions or in an IL-2-limited environment [32]. Epigenetic profiling of Tregs showed that most tTregs exhibit completely demethylated TSDR, whereas pTregs exhibit partially demethylated TSDR [33,34]. CNS1, on the other hand, is dispensable for the development of tTregs, but imperative for pTregs, which are strongly dependent on TGF-β signaling. CNS1 contains a TGF-β-NFAT response element [27]. The stability of pTreg and in vitro–derived Tregs (iTreg) may be dependent on acquiring both Foxp3 expression and Treg-specific DNA hypomethylated regions [35,36]. tTregs possess unique DNA hypomethylated features that are acquired during thymic Treg development and start before Foxp3 induction [20,21,33,37]. These features are imprinted in genes that are normally upregulated in unstimulated tTregs, such as *Foxp3*, *Ctla4*, *Tnfrsf18*, and *Ikzf2* [21]. 

Tregs can become dysregulated, and this has been linked to some autoimmune diseases. Tregs adoptively transferred into lymphopenic mice lose the expression of Foxp3 and gain effector characteristics [38]. Moreover, a subset of Tregs can lose the expression of Foxp3 under inflammatory settings, which can impact their immunosuppressive function [39,40]. Other Tregs expressing Tcon-lineage transcription factors and cytokines can still dampen the immune response and maintain immunological homeostasis [41]. In this review, we summarize the literature documenting the Tregs stability and plasticity, mostly based on mouse models, and we discuss the mechanisms underlying Treg instability and how this may apply to atherosclerosis. 

## 2. Phenotypic and Functional Adaptability of Tregs

**Figure 1:**
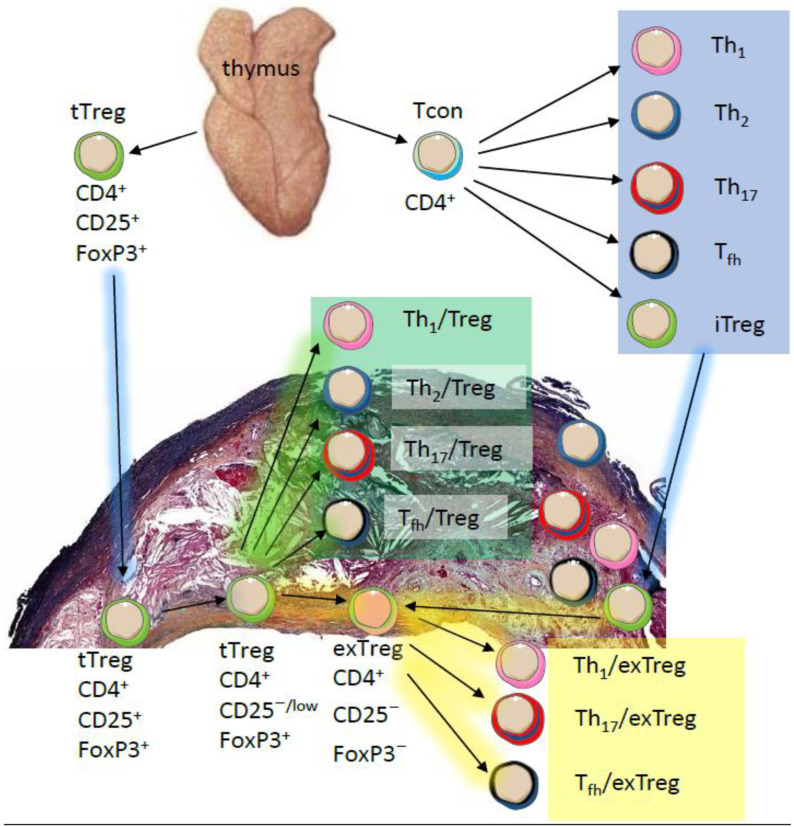
The thymus produces CD4^+^CD25^+^FoxP3^+^ tTregs (green), CD4^+^ conventional T cells (Tconv, blue) and many other cells (not shown). tTregs can migrate (blue arrows) into atherosclerotic lesions and the adventitia and maintain their phenotype. CD4^+^ Tconv differentiate into T-helper (Th)1, 2, and 17; T_fh_; and induced iTregs. All of these (blue box) can also migrate into atherosclerotic lesions and the adventitia. In the lesion environment or in secondary lymphoid organs (not shown), both tTregs and iTregs can gain expression of T-bet for Th_1_, GATA3 for Th_2_, RORγt for Th_17_ or Bcl6 for T_fh_, while maintaining expression of FoxP3 (Treg plasticity, green box). tTreg and iTregs can lose CD25 and FoxP3 expression and become exTregs that have a Th_1_, Th_17_, or T_fh_ phenotype (Treg instability, yellow box).

It is well established that most Tregs are stable and long-lasting under physiological conditions. However, several studies have challenged this notion and showed that prolonged exposure to inflammatory cues can promote Treg functional plasticity or affect Tregs stability [39,42,43]. Furthermore, IL-2 deprivation can create dysfunctional Tregs [44,45]. Moreover, metabolites and metabolic programs were shown to control Treg fate [46]. Instability and plasticity describe two distinct fates of Tregs. Instability describes the state at which Tregs lose Foxp3 expression and become so-called exTregs. This loss impairs the suppressive capacity (functionality) of these cells and allows them to acquire an effector-like phenotype. Loss of Foxp3 expression and gain of Th_1_, T_fh_, or Th_17_ effector phenotypes have been reported in different diseases such as rheumatoid arthritis [47] and atherosclerosis [42,48] (Figure 1). Moreover, the presence of “latent” Tregs among exTregs has been observed by one group [49,50]. These cells were shown to maintain the epigenetic Treg memory by retaining the demethylated status of CNS2 region and were shown to be able to revert to Foxp3^+^ Tregs upon TCR stimulation [49].

The term “plasticity” refers to the capacity of Tregs to acquire the migratory and functional characteristics of effector T cells while maintaining Foxp3 expression, so-called Th-like Tregs [44]. It can be envisioned that Treg plasticity is driving Treg heterogeneity in which multiple subsets of Th-like Tregs were reported. For example, IFNγ^+^ T-bet^+^CXCR3^+^ Th_1_-like Tregs, IL4^+^IL5^+^IL13^+^GATA3^+^ T_h_2-like Tregs, IL17A^+^RORγt^+^ Th_17_-like Tregs, and CXCR5^+^Bcl6^+^ICOS^+^PD1^+^ follicular Tregs have been identified under both physiological and pathological conditions [51,52,53,54,55]. It should be noted that, while instability is detrimental for Treg functionality, plasticity can be beneficial. It is thought that plasticity allows Tregs to adapt the transcriptional and migratory features of effector T cells, thus becoming more effective suppressors. For example, in response to IFNγ or IL-27 Tregs acquire Th_1_ characteristics by expressing T-bet and CXCR3, preferentially accumulate in Th_1_ inflammatory niches, and render Th1 cells more susceptible to suppression [41,56]. The same is true when Tregs acquire IRF4 expression and gain higher control over Th_2_ [55], or when Tregs acquire Bcl6 expression to control germinal center responses [57,58].

To evaluate Treg stability, multiple Treg-fate reporter mouse models have been generated [40,44,49] (Table 1). In all of these models, the Foxp3 promoter drives expression of a fluorescent protein (GFP) and/or Cre recombinase, using bacterial artificial chromosome (BAC)-derived transgenic or targeted knock-in mice. These mice were bred to reporter mice that harbor a transgene encoding loxP site-flanked stop codons in front of a reporter fluorescent protein (RFP) inserted into the Rosa26 locus. Thus, Foxp3 drives GFP and Cre, which removes the loxP sites, resulting in expression of RFP in cells that once expressed Foxp3, even if Foxp3-GFP-Cre expression is later lost. In such mice, current Tregs are yellow (GFP^+^RFP^+^), and ex-Tregs are red (GFP^−^RFP^+^). In a further refinement, GFP and Cre expression was made inducible by using a mutated form of human estrogen receptor (CreER^T2^; *Foxp3^GFP−CreERT2^*) [44]. In this system, in the absence of the ER^T2^ ligand tamoxifen, CreER^T2^ is sequestered in the cytoplasm. Ligand administration activates CreER^T2^, leading to its translocation to the nucleus, where Cre recombines the loxP sites. Endogenous mouse 17β-estradiol does not activate this form of ER mutant (ER^T2^); hereby, it eliminates the possibility of artifacts due to “leakiness” in expression [59]. 

Two of these models showed that 10–20% (depending on the lymphoid tissue) of the total Tregs were unstable and switched to form exTregs [40,49]. Moreover, the propensity of Treg switching increased in response to (chronic) inflammatory insults in these models. In the inducible model, the total Treg lineage was shown to be remarkably stable with less than 5% of unstable peripheral Treg (exTregs). In this model, exposing Tregs to inflammatory insults did not undermine their stability. However, this model may underestimate the switched population, because only the Tregs that had been labeled during Tamoxifen injection are tracked. The cells that switched after tamoxifen injection are not labeled.

The reason for these divergent observations is likely, at least in part, technical. In the first two models, Tregs are labeled from birth to the time of data collection. In the inducible model, Tregs are labeled at the time of induction with Tamoxifen. This resembles taking a snapshot within a limited time period during Treg development. Indeed, the model proposed by Miyao and colleagues showed that exTregs appeared during ontogeny and accumulated through adulthood. In addition, pTregs contain an unstable pool of Tregs [50,60]. Thus, it is possible that the labeling system in the Rubstov et al. model is less representative of the unstable pTreg pool, which could have been accumulated from childhood through adulthood. Indeed, in a recent tTreg tracing model, in which CNS-1 was deleted from Foxp3 locus to limit the tracing to mature tTregs, only ~1% of tTregs lost Foxp3 expression under physiological conditions [61]. In this model, it has been shown that activated tTregs (CD62L^lo^CD44^hi^CCR7^lo^) are more likely to lose Foxp3 activity than resting/central Tregs (CD62L^hi^CD44^lo^CCR7^hi^). Another factor to consider when comparing Treg-fate reporter models is the type/intensity of inflammatory triggers that were utilized to challenge Treg stability. It appears that IL-2 and TCR engagement provide stability signals, while pro-inflammatory cytokines like IL-6, IL-4, and IL-12 can render Tregs unstable [39,62,63,64]. Collectively, despite the discrepancies in the percentage of the observed exTregs, the lineage tracing models portray the Treg pool as a stable cell-type of CD4 T cells, with a minor unstable subset, at least under the conditions studied so far.

## 3. Possible Mechanisms of Treg Instability

Both cell-intrinsic (TCR) and extrinsic (pro-inflammatory cues) factors modulate the Treg program. However, to what extent each is correlated to Treg stability is unknown. Two leading hypotheses may explain Treg instability. The first suggests that TCR stimulation may prime the epigenome of developing thymocytes to develop toward Tregs. The second hypothesis suggests that proinflammatory cytokines such as IL-6 and IL-4 may be major instability-inducing factors in mature Tregs. 

Optimal Treg development depends on both TCR signal intensity and duration. The strength of TCR signal during positive and negative selection may follow the Goldilocks principle: Too strong results in clonal deletion, and too weak prevents Foxp3 induction [9,65,66]. Phosphoinositol 3′-kinase (PI(3)K)/Akt signaling downstream of TCR activation is less active in Tregs than Tcons [67,68]. Activation of Akt strongly represses Foxp3 induction during tTreg development as well as during iTreg formation [68]. The duration of TCR signaling along with the right co-stimulators appears to control the Treg-specific DNA hypomethylation. For example, constitutive or repetitive TCR stimulation downregulates Foxp3 in both mature activated tTregs and iTregs [67,69]. Premature termination of TCR signaling and PI3k and mTOR inhibition induces Foxp3 expression from CD4^+^CD25^−^CD62L^hi^ cells by changing the methylation status of the *Foxp3* locus [67]. In a recent study, the absence of anti-CD28 during the induction of iTregs from CD4^+^CD25^−^CD62L^hi^ resulted in more epigenetically stable Tregs [37]. Overall, stably committed Tregs require a precise amount of TCR strength (for Foxp3 induction) and duration (for DNA-hypomethylation). Thus, effector Tregs may lose their suppressive functionality by interacting with ligand(s) similar to their cognate self-antigen that binds the TCR with higher affinity. Such interactions may lead to strong ICOS signaling that activates the PI3k/AKT signaling pathway, thus potentiating Treg instability [70]. Although both effector and resting Tregs receive continuous TCR signaling, the signal intensity skews the Treg phenotype: With a strong TCR signal, Tregs adopt an activated phenotype in which Treg homeostasis and function depend on ICOS signaling rather than IL-2 [71,72]. Supporting this hypothesis, a recent study has shown that highly self-reactive Tregs are more susceptible to upregulate T-bet and CXCR3 in response to TCR signaling rather than environmental cues [73]. However, in this study, the Tregs were stable, maintained their DNA-hypomethylation and did not express IFNγ. It is unclear how TCR signal duration and the type of co-stimulators maintain the epigenetic features of Tregs. 

Under inflammatory conditions, the imbalance between local pro-inflammatory cytokines and Treg survival factors like IL-2 could impair Treg stability. Several studies have shown that IL-6, IL-4, IL-12, and IL-32 drive the inactivation of Foxp3 expression [39,74,75,76,77]. On the other hand, lack of IL-2 in diabetic islets [78] and under highly polarized Th_1_ immune responses [43] reduced Treg numbers and compromised their function. Treatment with IL-2 and anti-IL-2 complexes stabilized Foxp3 expression during EAE and expanded the Treg population in clinical trials [79,80]. 

Recent studies have demonstrated crucial roles for nutrients, metabolites, and cellular metabolism in modulating Treg functions. Vitamins such as A, D, and C can promote Foxp3 expression mainly by enhancing CNS demethylation [28,81,82,83]. Metabolites such as tryptophan induce Foxp3 expression by decreasing IL-6 production and increasing TGF-β expression in dendritic cells [84]. Other metabolites such as extracellular NAD^+^ and ATP can induce Treg conversion to Th_17_ cells by stimulating purinergic 2X receptor signaling, which induces T-cell activation, proliferation, and apoptosis [85,86]. Cellular metabolism can also modulate Foxp3 expression. The metabolic status is affected by the type of activated signaling pathway(s). Activation of the PI3K and Akt and mammalian target of rapamycin (mTOR) pathway promotes glycolysis. The AMP-activated protein kinase (AMPK) pathway promotes oxidative phosphorylation (OXPHOS) and fatty acid oxidation (FAO) [87]. Unlike Tcons, Tregs prefer OXPHOS and FAO to glycolysis [88]. Studies have shown that Foxp3 induction promotes OXPHOS over glycolysis by suppressing the transcription of Myc and the glucose transporter Glut-1, and by inhibiting the PI(3)K and Akt and mTOR pathway [89,90]. Conversely, unrestrained glycolysis induces destabilization of Tregs [91,92,93]. Treg-specific deletion of PTEN (phosphatase and tensin homolog), a negative regulator of the glycolysis-promoting PI(3)K-Akt-mTORC2 pathway, promotes phenotypic transition of Tregs, which is characterized by a loss of their surface marker interleukin-2 receptor α subunit (CD25) and, ultimately, their lineage defining factor, Foxp3. Concomitantly, PTEN-deficient Tregs lose their suppressive capacity and the resulting overactivation of Th_1_ and T_fh_ responses gives rise to an autoimmune-lymphoproliferative disease [93]. Tregs of transgenic mice harboring T-cell-specific expression of constitutively active Glut1, likewise, lose Foxp3 expression, as well as their suppressive capacity [90].

On the other hand, disrupting OXPHOS by ablation of mitochondrial transcription factor A or mitochondrial complex III impairs Treg suppressive function without altering Foxp3 stability [94,95]. Lipid metabolism can also enhance Treg functionality. LKB-1, a key player in lipid metabolism in T cells, stabilizes Foxp3 expression by inhibiting STAT4-mediated CNS2 methylation [96]. Additionally, LKB-1 regulates the mevalonate pathway, related to intracellular cholesterol homeostasis, and thereby inhibits inflammatory cytokine production in Tregs and maintains their suppressiveness [97]. The mevalonate pathway can also be regulated by raptor, an essential component of mTORC1. Deletion of raptor impairs Treg function without affecting Foxp3 expression [98]. Interestingly, the mevalonate pathway is strongly inhibited by statins, a class of cholesterol-lowering drugs [99]. A recent study showed that the mTOR pathway can promote Treg stability [100], possibly because mTOR deficiency upregulates the levels of glutaminolysis and α-ketoglutarate, resulting in partial remethylation of the CNS2 region of *Foxp3* [100,101,102]. 

## 4. Treg Adaptability in Atherosclerosis

In atherosclerosis, both Treg instability and Treg plasticity were reported (Figure 1). In 2016, Li et al. fed Apoe^−/−^ mice on a Western diet (WD) for 12–20 weeks, to report the presence of plastic Tregs in aortas. These cells expressed IFNγ, T-bet, and Foxp3, were CD25^low^ or negative, and accounted for 80% of all CCR5^+^ αβ T cells. The percentage of this population in the para-aortic lymph nodes (paLN) was significantly less (~15% of all CCR5^+^ αβ T cells) [48], and these cells were undetectable in the spleen. A similar population was observed by Butcher at al. [42]. In their study, IFNγ-producing Foxp3^+^ cells (Th_1_/Tregs) were evident in aortas (15% of total CD4 T cells) of aged *Apoe*^−/−^ mice after two weeks of WD. This population was observed in both spleen and paLNs although at lower percentages (~12% in spleen and ~4% in paLNs). This work established CCR5 as an extracellular marker for Th_1_/Tregs. Previous studies have shown IFNγ to be important in generating atheroprotective CXCR3^+^ Tregs [41]. The authors tested the suppressive capacity of atherosclerotic Th_1_/Tregs and found them to be non-suppressive. In their efforts to track the source of Th_1_/Tregs, the authors injected Tregs from fate-reporter mice, *Foxp3^yfp-cre/yfp-cre^Rosa26R^tdTomato/tdTomato^*, into aged *Apoe*^−/−^ mice. After two weeks of a Western diet, 30% of the injected cells were Foxp3^+^ CCR5^+^, suggesting that plastic Tregs arise from Tregs. In this system, however, none of the transferred Tregs lost the Foxp3-YFP signal, suggesting that two weeks of a WD is not enough to abolish Foxp3 expression and cause Treg instability.

In vitro, prolonged exposure to inflammatory cytokines like IL-12, IL-27, and IFNγ promoted Treg plasticity, suggesting that such cytokines could drive Treg plasticity in atherosclerosis. Indeed, IL-12 induces the formation of IFNγ^+^-Tregs by directly activating the PI(3)-AKT and Foxo1/3 pathway [103]. Foxo1/3 phosphorylation by AKT results in their cytoplasmic sequestration, preventing the interaction with their transcriptional targets. Foxo1 upregulates Foxp3 and *Ctla4* gene expression and dampens *Ifng* expression [104,105,106]. In addition, decreased Foxo1 activity correlates with decreased glycolysis. This induce Tregs to acquire Th_1_-like program. As mentioned earlier, the activation of PI(3)K and AKT metabolic pathway can upregulate Glut-1, which can promote glycolysis and induce Treg plasticity [93,107]. To further support the atherogenic role of Th_1_/Treg in atherosclerosis, the authors used a Th_1_/Treg-prone mouse model (*Mir146a*^−/−^ mice). Moreover, miR-146a regulates the activation of Stat1 in Treg cells, which maintains efficient control of a spontaneous IFNγ-dependent Th_1_ response and prevents the conversion of activated Tregs to IFNγ-producing Th1-like cells [108]. When *Mir146a*^−/−^ Tregs (CD4^+^CD25^+^) were transferred into 27-week-old *Apoe*^−/−^ mice for eight weeks, these mice developed more plaques than the mice receiving Mir146 sufficient Tregs. However, the lesion percentage was similar between mice that received Tregs from *Mir146a*^−/−^ and sham-treated mice (baseline). This suggests that Th_1_/Treg-prone cells do not cause inflammation per se; instead, they fail to suppress atherogenic adaptive immune responses. Overall, this study reported, for the first time, non-suppressive plastic Th_1_/Tregs in atherosclerosis and proved their atherogenicity. 

In another study, Treg-fate reporter mice (*Foxp3^YFP^*^-*Cre*^*Rosa26^RFP^ApoE*^−/−^) showed Treg instability and exTreg formation in atherosclerosis [109]. In this study, exTregs were evident in the paLNs, spleen, and aorta of mice fed either chow diet or WD (15 weeks), but the proportion was higher under WD. Interrogation of exTreg phenotype(s) by using intracellular cytokine staining (ICS) suggested that Tregs can switch to Th_1_ and atherogenic T_fh_ under atherosclerotic conditions. Moreover, flow cytometry analysis showed that exTregs display low CD25 expression and increased IL-6Rα when compared with Tregs. The conversion of Tregs to T_fh_ may be caused by the disruption of intracellular cholesterol homeostasis in WD-fed *ApoE*^−/−^ mice, because subcutaneous injections of ApoAI, a necessary component for the formation of nascent HDL particles, prompted cholesterol efflux, and sustained Treg stability. Interestingly, ApoAI treatment reduced T_fh_ exTregs, but not Th_1_ exTregs, suggesting different mechanisms underlying Treg conversion to Th_1_. Overall, this work illustrates how the hypercholesterolemic environment may shape the Tregs fate. 

Recent work from our lab demonstrated that prolonged exposure to a hypercholesterolemic environment promotes the conversion of apolipoprotein B (ApoB) antigen-specific Tregs to atherogenic Th_17_ and Th_1_-like cells [110]. Time course scRNA-seq data analysis of ApoB-specific T cells demonstrated a phenotypic switch from Foxp3 expression toward a novel subpopulation of T-bet^+^RORγt^+^ cells in chow diet (CD)- fed *ApoE*^−/−^ mice. Moreover, Tregs transferred into old *ApoE*^−/−^ mice lost Foxp3 expression and gained T-bet or RORγt. These results were supported by an increase of Th_1_ and Th_17_ cells in WD-fed *ApoE*^−/−^ mice in comparison to CD fed *ApoE*^−/−^ mice. Gain of RORγt expression in Foxp3^+^ cells appears to be driven by Treg exposure to proinflammatory cytokines such as IL-6 and IL-1β. Indeed, we observed an upregulation of these cytokines in 20-week-old *ApoE*^−/−^ mice. Moreover, IL-6, along with TGF-β, induces c-Maf in Th_17_ cells [111]. Indeed, c-Maf was upregulated in Th_17_ cells. Interestingly, in the intestinal niche, c-Maf promotes RORγt^+^ transcription in protective Foxp3^+^ cells in response to microbiota [112]. Another player in Treg conversion toward RORγt^+^ is continuous antigen exposure. Moreover, oxLDL, a known autoantigen in atherosclerosis that contains ApoB, is known to accumulate in atherosclerotic plaques, where it may reduce Treg stability and function [113,114]. A recent study showed that hypercholesterolemia induces hepatic iTreg/Th_17_ formation. These cells were shown to be home to aortas [115]. These cells may lose Foxp3 under atherosclerotic conditions and become atherogenic. Hypoxia is common in atherosclerotic plaques [116]. Previous studies have shown that hypoxia-inducible factor-1α (HIF-1α) induces RORγt expression and prevents iTreg formation by inducing Foxp3 proteasomal degradation [117]. Augmented HIF-1α activity in Tregs can promote glycolysis and induce the formation of IFN-γ^+^ Th_1_-like Tregs [118]. CNS2 demethylation is dependent on oxygen availability. The ten-eleven translocases TET2 and TET3 that mediate CNS2 demethylation require molecular oxygen (O_2_) for their activity [119]. How hypoxia affects the fate of Tregs in atherosclerosis is not known.

Taken together, the functional and phenotypic reprogramming of Tregs in atherosclerosis is not fully understood. Some results point to a loss of Treg stability, while others point to Treg plasticity. The discrepancies could be due to different time windows during which the Tregs were under inflammatory pressure. In the Butcher et al. study, the Treg-fate tracer mice were on a wild-type (*ApoE*^+/+^) background; thus; exTreg formation during atherogenesis was not investigated. The Treg fate was assessed two weeks after adoptive transfer to atherosclerotic *ApoE*^−/−^ mice. On the other side, Gaddis et al. had Treg-fate tracer mice crossed into the *ApoE*^−/−^ background. Thereby, Tregs were under inflammatory conditions during the entire study. It is noteworthy to mention that the study by Gaddis et al. does not rule out the availability of plastic Treg in atherosclerosis. The percentage of IFNγ^+^ -producing Tregs, which contain Th_1_/Tregs, was not reported. 

## 5. Conclusions

Although Tregs are a largely stable lineage, recent studies have shown that the Treg program is mutable and that Tregs can lose their function, along with their lineage-marker (Foxp3), to form pathogenic exTregs. Emerging evidence indicates that atherosclerotic *Apoe*^−/−^ mice have non-suppressive Th1-like Tregs [42] and that Tregs can convert to pathogenic Tfh and Th1 and Th17-like cells under atherosclerotic conditions [109,110]. However, the impact of naturally formed exTreg (from pTregs or tTregs) on the development of atherosclerosis remains to be elucidated. To date, the proatherogenic properties of exTregs have not been tested in rigorous adoptive transfer experiments. It remains unclear whether the exTregs formed in atherosclerotic mice are “latent” Tregs and can revert to fully functional Tregs when appropriately stimulated, in the absence of proinflammatory cytokines and the presence of a cognate self-antigen such as ApoB. Tolerogenic vaccines against atherosclerosis can expand atheroprotective Tregs and prevent atherosclerosis [120,121], but there is no evidence that a tolerogenic vaccine works in mice with established atherosclerosis (therapeutic vaccine). Since Treg function and stability is dependent on TCR signaling, metabolic processes, and cytokine signaling, the atherosclerotic environment has a lot to offer to drive Treg instability. Hypercholesterolemia, hypoxia, and enrichment of proinflammatory cytokines are all candidates for disrupting the Treg program and promote their conversion to exTregs. 

## Figures and Tables

**Table 1:** Foxp3 lineage tracking mouse models.

Foxp3 Allele	IRES for Reporter/Cre	Allele Type	ROSA-Locus	Inducible	Advantages	Limitations	References
Foxp3-EGFP-Cre	No	Transgenic (BAC), Recombinase-expressing, Reporter	Rosa26-loxP-Stop-loxP-YFP	No	Natural FoxP3 locus intact	May overestimate the switched population. BAC transgene inactivation in some Foxp3^+^ cells has been reported. Promoter may be incomplete. Enhancers may be missing.	[31]
Foxp3-IRES-GFP-iCre	Yes	Targeted (knock-in), Recombinase-expressing, Reporter	Rosa26-loxP-Stop-loxP-RFP	No	Reporter expressed under the natural FoxP3 promoter with enhancers intact.	May overestimate the switched population. Foxp3 activation can occur early during development in non-Treg cells. In this mouse, Cre activity was reported in CD8 T cells, B cells, and myeloid cells.	[40]
Foxp3-IRES-*EGFP-Cre-ERT2*	Yes	Targeted (knock-in), Recombinase-expressing, Reporter, Inducible	Rosa26-loxP-Stop-loxP-YFP	Yes, with Tamoxifen	Reporter expressed under the natural FoxP3 promoter with enhancers intact.	May underestimate the switched population. This mouse tracks only the Tregs that were labeled during Tamoxifen injection. If the switched subset developed after tamoxifen injection, it will not be visible.	[35]
Foxp3-dCNS1-hCre-2A-eqFP650-2A-Thy1.1	No	Transgenic (BAC), Recombinase-expressing, Reporter	Rosa26-loxP-Stop-loxP-YFP	No	This mouse detects the fate of tTregs but not pTregs. In YFP+ cells Thy1.1 is lost before Foxp3, making Thy1.1 a marker for cells that are about to lose Foxp3 expression.	In this model, to define exTregs, researchers need to stain for intracellular Foxp3.	[60]

BAC, bacterial artificial chromosome

## References

[1] Gistera A., Hansson G.K. (2017). The immunology of atherosclerosis. Nat. Rev. Nephrol..

[2] Ridker P.M., Everett B.M., Thuren T., MacFadyen J.G., Chang W.H., Ballantyne C., Fonseca F., Nicolau J., Koenig W., Anker S.D. (2017). Antiinflammatory Therapy with Canakinumab for Atherosclerotic Disease. N. Engl. J. Med..

[3] Tardif J.C., Kouz S., Waters D.D., Bertrand O.F., Diaz R., Maggioni A.P., Pinto F.J., Ibrahim R., Gamra H., Kiwan G.S. (2019). Efficacy and Safety of Low-Dose Colchicine after Myocardial Infarction. N. Engl. J. Med..

[4] Glass C.K., Witztum J.L. (2001). Atherosclerosis. The road ahead. Cell.

[5] Wolf D., Ley K. (2019). Immunity and Inflammation in Atherosclerosis. Circ. Res..

[6] Ley K. (2020). Role of the adaptive immune system in atherosclerosis. Biochem. Soc. Trans..

[7] Saigusa R., Winkels H., Ley K. (2020). T cell subsets and functions in atherosclerosis. Nat. Rev. Cardiol..

[8] Ou H.X., Guo B.B., Liu Q., Li Y.K., Yang Z., Feng W.J., Mo Z.C. (2018). Regulatory T cells as a new therapeutic target for atherosclerosis. Acta Pharm. Sin..

[9] Jordan M.S., Boesteanu A., Reed A.J., Petrone A.L., Holenbeck A.E., Lerman M.A., Naji A., Caton A.J. (2001). Thymic selection of CD4^+^CD25^+^ regulatory T cells induced by an agonist self-peptide. Nat. Immunol..

[10] Wei S., Kryczek I., Zou W. (2006). Regulatory T-cell compartmentalization and trafficking. Blood.

[11] Zheng S.G., Wang J.H., Gray J.D., Soucier H., Horwitz D.A. (2004). Natural and induced CD4^+^CD25^+^ cells educate CD4^+^CD25^−^ cells to develop suppressive activity: The role of IL-2, TGF-beta, and IL-10. J. Immunol..

[12] Yamagiwa S., Gray J.D., Hashimoto S., Horwitz D.A. (2001). A role for TGF-beta in the generation and expansion of CD4^+^CD25^+^ regulatory T cells from human peripheral blood. J. Immunol..

[13] Zheng S.G., Wang J., Wang P., Gray J.D., Horwitz D.A. (2007). IL-2 is essential for TGF-beta to convert naive CD4^+^CD25^−^ cells to CD25^+^Foxp3^+^ regulatory T cells and for expansion of these cells. J. Immunol..

[14] Chen W., Jin W., Hardegen N., Lei K.J., Li L., Marinos N., McGrady G., Wahl S.M. (2003). Conversion of peripheral CD4^+^CD25^−^ naive T cells to CD4^+^CD25^+^ regulatory T cells by TGF-beta induction of transcription factor Foxp3. J. Exp. Med..

[15] Carrier Y., Yuan J., Kuchroo V.K., Weiner H.L. (2007). Th3 cells in peripheral tolerance. I. Induction of Foxp3-positive regulatory T cells by Th3 cells derived from TGF-beta T cell-transgenic mice. J. Immunol..

[16] Seddiki N., Santner-Nanan B., Martinson J., Zaunders J., Sasson S., Landay A., Solomon M., Selby W., Alexander S.I., Nanan R. (2006). Expression of interleukin (IL)-2 and IL-7 receptors discriminates between human regulatory and activated T cells. J. Exp. Med..

[17] Khattri R., Cox T., Yasayko S.A., Ramsdell F. (2017). Pillars Article: An Essential Role for Scurfin in CD4^+^CD25^+^ T Regulatory Cells. J. Immunol..

[18] Fontenot J.D., Gavin M.A., Rudensky A.Y. (2017). Pillars Article: Foxp3 Programs the Development and Function of CD4^+^CD25^+^ Regulatory T Cells. J. Immunol..

[19] Wildin R.S., Smyk-Pearson S., Filipovich A.H. (2002). Clinical and molecular features of the immunodysregulation, polyendocrinopathy, enteropathy, X linked (IPEX) syndrome. J. Med. Genet..

[20] Sugimoto N., Oida T., Hirota K., Nakamura K., Nomura T., Uchiyama T., Sakaguchi S. (2006). Foxp3-dependent and -independent molecules specific for CD25^+^CD4^+^ natural regulatory T cells revealed by DNA microarray analysis. Int. Immunol..

[21] Ohkura N., Hamaguchi M., Morikawa H., Sugimura K., Tanaka A., Ito Y., Osaki M., Tanaka Y., Yamashita R., Nakano N. (2012). T cell receptor stimulation-induced epigenetic changes and Foxp3 expression are independent and complementary events required for Treg cell development. Immunity.

[22] Gavin M.A., Rasmussen J.P., Fontenot J.D., Vasta V., Manganiello V.C., Beavo J.A., Rudensky A.Y. (2007). Foxp3-dependent programme of regulatory T-cell differentiation. Nature.

[23] Hill J.A., Feuerer M., Tash K., Haxhinasto S., Perez J., Melamed R., Mathis D., Benoist C. (2007). Foxp3 transcription-factor-dependent and -independent regulation of the regulatory T cell transcriptional signature. Immunity.

[24] Groux H., O’Garra A., Bigler M., Rouleau M., Antonenko S., de Vries J.E., Roncarolo M.G. (1997). A CD4^+^ T-cell subset inhibits antigen-specific T-cell responses and prevents colitis. Nature.

[25] Zheng Y., Josefowicz S.Z., Kas A., Chu T.T., Gavin M.A., Rudensky A.Y. (2007). Genome-wide analysis of Foxp3 target genes in developing and mature regulatory T cells. Nature.

[26] Williams L.M., Rudensky A.Y. (2007). Maintenance of the Foxp3-dependent developmental program in mature regulatory T cells requires continued expression of Foxp3. Nat. Immunol..

[27] Zheng Y., Josefowicz S., Chaudhry A., Peng X.P., Forbush K., Rudensky A.Y. (2010). Role of conserved non-coding DNA elements in the Foxp3 gene in regulatory T-cell fate. Nature.

[28] Yue X., Trifari S., Aijo T., Tsagaratou A., Pastor W.A., Zepeda-Martinez J.A., Lio C.W., Li X., Huang Y., Vijayanand P. (2016). Control of Foxp3 stability through modulation of TET activity. J. Exp. Med..

[29] Li X., Liang Y., LeBlanc M., Benner C., Zheng Y. (2014). Function of a Foxp3 cis-element in protecting regulatory T cell identity. Cell.

[30] Soper D.M., Kasprowicz D.J., Ziegler S.F. (2007). IL-2Rbeta links IL-2R signaling with Foxp3 expression. Eur. J. Immunol..

[31] Burchill M.A., Yang J., Vogtenhuber C., Blazar B.R., Farrar M.A. (2007). IL-2 receptor beta-dependent STAT5 activation is required for the development of Foxp3^+^ regulatory T cells. J. Immunol..

[32] Feng Y., Arvey A., Chinen T., van der Veeken J., Gasteiger G., Rudensky A.Y. (2014). Control of the inheritance of regulatory T cell identity by a cis element in the Foxp3 locus. Cell.

[33] Floess S., Freyer J., Siewert C., Baron U., Olek S., Polansky J., Schlawe K., Chang H.D., Bopp T., Schmitt E. (2007). Epigenetic control of the foxp3 locus in regulatory T cells. PLoS Biol..

[34] Baron U., Floess S., Wieczorek G., Baumann K., Grutzkau A., Dong J., Thiel A., Boeld T.J., Hoffmann P., Edinger M. (2007). DNA demethylation in the human FOXP3 locus discriminates regulatory T cells from activated FOXP3^+^ conventional T cells. Eur. J. Immunol..

[35] Ohkura N., Sakaguchi S. (2020). Transcriptional and epigenetic basis of Treg cell development and function: Its genetic anomalies or variations in autoimmune diseases. Cell Res..

[36] Helmin K.A., Morales-Nebreda L., Torres Acosta M.A., Anekalla K.R., Chen S.Y., Abdala-Valencia H., Politanska Y., Cheresh P., Akbarpour M., Steinert E.M. (2020). Maintenance DNA methylation is essential for regulatory T cell development and stability of suppressive function. J. Clin. Investig..

[37] Mikami N., Kawakami R., Chen K.Y., Sugimoto A., Ohkura N., Sakaguchi S. (2020). Epigenetic conversion of conventional T cells into regulatory T cells by CD28 signal deprivation. Proc. Natl. Acad. Sci. USA.

[38] Duarte J.H., Zelenay S., Bergman M.L., Martins A.C., Demengeot J. (2009). Natural Treg cells spontaneously differentiate into pathogenic helper cells in lymphopenic conditions. Eur. J. Immunol..

[39] Kastner L., Dwyer D., Qin F.X. (2010). Synergistic effect of IL-6 and IL-4 in driving fate revision of natural Foxp3^+^ regulatory T cells. J. Immunol..

[40] Zhou X., Bailey-Bucktrout S.L., Jeker L.T., Penaranda C., Martinez-Llordella M., Ashby M., Nakayama M., Rosenthal W., Bluestone J.A. (2009). Instability of the transcription factor Foxp3 leads to the generation of pathogenic memory T cells in vivo. Nat. Immunol..

[41] Koch M.A., Tucker-Heard G., Perdue N.R., Killebrew J.R., Urdahl K.B., Campbell D.J. (2009). The transcription factor T-bet controls regulatory T cell homeostasis and function during type 1 inflammation. Nat. Immunol..

[42] Butcher M.J., Filipowicz A.R., Waseem T.C., McGary C.M., Crow K.J., Magilnick N., Boldin M., Lundberg P.S., Galkina E.V. (2016). Atherosclerosis-Driven Treg Plasticity Results in Formation of a Dysfunctional Subset of Plastic IFNgamma+ Th1/Tregs. Circ. Res..

[43] Oldenhove G., Bouladoux N., Wohlfert E.A., Hall J.A., Chou D., Dos Santos L., O’Brien S., Blank R., Lamb E., Natarajan S. (2009). Decrease of Foxp3^+^ Treg cell number and acquisition of effector cell phenotype during lethal infection. Immunity.

[44] Rubtsov Y.P., Niec R.E., Josefowicz S., Li L., Darce J., Mathis D., Benoist C., Rudensky A.Y. (2010). Stability of the regulatory T cell lineage in vivo. Science.

[45] Setoguchi R., Hori S., Takahashi T., Sakaguchi S. (2005). Homeostatic maintenance of natural Foxp3^+^ CD25^+^ CD4^+^ regulatory T cells by interleukin (IL)-2 and induction of autoimmune disease by IL-2 neutralization. J. Exp. Med..

[46] Shi H., Chi H. (2019). Metabolic Control of Treg Cell Stability, Plasticity, and Tissue-Specific Heterogeneity. Front. Immunol..

[47] Komatsu N., Okamoto K., Sawa S., Nakashima T., Oh-hora M., Kodama T., Tanaka S., Bluestone J.A., Takayanagi H. (2014). Pathogenic conversion of Foxp3^+^ T cells into TH17 cells in autoimmune arthritis. Nat. Med..

[48] Li J., McArdle S., Gholami A., Kimura T., Wolf D., Gerhardt T., Miller J., Weber C., Ley K. (2016). CCR5^+^T-bet^+^FoxP3^+^ Effector CD4 T Cells Drive Atherosclerosis. Circ. Res..

[49] Miyao T., Floess S., Setoguchi R., Luche H., Fehling H.J., Waldmann H., Huehn J., Hori S. (2012). Plasticity of Foxp3^+^ T cells reflects promiscuous Foxp3 expression in conventional T cells but not reprogramming of regulatory T cells. Immunity.

[50] Komatsu N., Mariotti-Ferrandiz M.E., Wang Y., Malissen B., Waldmann H., Hori S. (2009). Heterogeneity of natural Foxp3^+^ T cells: A committed regulatory T-cell lineage and an uncommitted minor population retaining plasticity. Proc. Natl. Acad. Sci. USA.

[51] Tan T.G., Mathis D., Benoist C. (2016). Singular role for T-BET^+^CXCR3^+^ regulatory T cells in protection from autoimmune diabetes. Proc. Natl. Acad. Sci. USA.

[52] Bovenschen H.J., van de Kerkhof P.C., van Erp P.E., Woestenenk R., Joosten I., Koenen H.J. (2011). Foxp3^+^ regulatory T cells of psoriasis patients easily differentiate into IL-17A-producing cells and are found in lesional skin. J. Investig. Derm..

[53] Qiu R., Zhou L., Ma Y., Zhou L., Liang T., Shi L., Long J., Yuan D. (2020). Regulatory T Cell Plasticity and Stability and Autoimmune Diseases. Clin. Rev. Allergy Immunol..

[54] Wollenberg I., Agua-Doce A., Hernandez A., Almeida C., Oliveira V.G., Faro J., Graca L. (2011). Regulation of the germinal center reaction by Foxp3^+^ follicular regulatory T cells. J. Immunol..

[55] Zheng Y., Chaudhry A., Kas A., deRoos P., Kim J.M., Chu T.T., Corcoran L., Treuting P., Klein U., Rudensky A.Y. (2009). Regulatory T-cell suppressor program co-opts transcription factor IRF4 to control T(H)2 responses. Nature.

[56] Hall A.O., Beiting D.P., Tato C., John B., Oldenhove G., Lombana C.G., Pritchard G.H., Silver J.S., Bouladoux N., Stumhofer J.S. (2012). The cytokines interleukin 27 and interferon-gamma promote distinct Treg cell populations required to limit infection-induced pathology. Immunity.

[57] Linterman M.A., Pierson W., Lee S.K., Kallies A., Kawamoto S., Rayner T.F., Srivastava M., Divekar D.P., Beaton L., Hogan J.J. (2011). Foxp3^+^ follicular regulatory T cells control the germinal center response. Nat. Med..

[58] Chung Y., Tanaka S., Chu F., Nurieva R.I., Martinez G.J., Rawal S., Wang Y.H., Lim H., Reynolds J.M., Zhou X.H. (2011). Follicular regulatory T cells expressing Foxp3 and Bcl-6 suppress germinal center reactions. Nat. Med..

[59] Feil R., Wagner J., Metzger D., Chambon P. (1997). Regulation of Cre recombinase activity by mutated estrogen receptor ligand-binding domains. Biochem. Biophys. Res. Commun..

[60] Josefowicz S.Z., Niec R.E., Kim H.Y., Treuting P., Chinen T., Zheng Y., Umetsu D.T., Rudensky A.Y. (2012). Extrathymically generated regulatory T cells control mucosal TH2 inflammation. Nature.

[61] Zhang Z., Zhang W., Guo J., Gu Q., Zhu X., Zhou X. (2017). Activation and Functional Specialization of Regulatory T Cells Lead to the Generation of Foxp3 Instability. J. Immunol..

[62] Barbi J., Pardoll D., Pan F. (2014). Treg functional stability and its responsiveness to the microenvironment. Immunol. Rev..

[63] Guo H., Xun L., Zhang R., Hu F., Luan J., Lao K., Wang X., Gou X. (2019). Stability and inhibitory function of Treg cells under inflammatory conditions in vitro. Exp. Ther. Med..

[64] Zhao J., Zhao J., Perlman S. (2012). Differential effects of IL-12 on Tregs and non-Treg T cells: Roles of IFN-gamma, IL-2 and IL-2R. PLoS ONE.

[65] Kawahata K., Misaki Y., Yamauchi M., Tsunekawa S., Setoguchi K., Miyazaki J., Yamamoto K. (2002). Generation of CD4^+^CD25^+^ regulatory T cells from autoreactive T cells simultaneously with their negative selection in the thymus and from nonautoreactive T cells by endogenous TCR expression. J. Immunol..

[66] Gabrysova L., Christensen J.R., Wu X., Kissenpfennig A., Malissen B., O’Garra A. (2011). Integrated T-cell receptor and costimulatory signals determine TGF-beta-dependent differentiation and maintenance of Foxp3^+^ regulatory T cells. Eur. J. Immunol..

[67] Sauer S., Bruno L., Hertweck A., Finlay D., Leleu M., Spivakov M., Knight Z.A., Cobb B.S., Cantrell D., O’Connor E. (2008). T cell receptor signaling controls Foxp3 expression via PI3K, Akt, and mTOR. Proc. Natl. Acad. Sci. USA.

[68] Haxhinasto S., Mathis D., Benoist C. (2008). The AKT-mTOR axis regulates de novo differentiation of CD4^+^Foxp3^+^ cells. J. Exp. Med..

[69] Hoffmann P., Boeld T.J., Eder R., Huehn J., Floess S., Wieczorek G., Olek S., Dietmaier W., Andreesen R., Edinger M. (2009). Loss of FOXP3 expression in natural human CD4^+^CD25^+^ regulatory T cells upon repetitive in vitro stimulation. Eur. J. Immunol..

[70] Zhang Z., Zhou X. (2019). Foxp3 Instability Helps tTregs Distinguish Self and Non-self. Front. Immunol..

[71] Smigiel K.S., Richards E., Srivastava S., Thomas K.R., Dudda J.C., Klonowski K.D., Campbell D.J. (2014). CCR7 provides localized access to IL-2 and defines homeostatically distinct regulatory T cell subsets. J. Exp. Med..

[72] Zemmour D., Zilionis R., Kiner E., Klein A.M., Mathis D., Benoist C. (2018). Single-cell gene expression reveals a landscape of regulatory T cell phenotypes shaped by the TCR. Nat. Immunol..

[73] Sprouse M.L., Scavuzzo M.A., Blum S., Shevchenko I., Lee T., Makedonas G., Borowiak M., Bettini M.L., Bettini M. (2018). High self-reactivity drives T-bet and potentiates Treg function in tissue-specific autoimmunity. JCI Insight.

[74] Bettelli E., Carrier Y., Gao W., Korn T., Strom T.B., Oukka M., Weiner H.L., Kuchroo V.K. (2006). Reciprocal developmental pathways for the generation of pathogenic effector TH17 and regulatory T cells. Nature.

[75] Zheng S.G., Wang J., Horwitz D.A. (2008). Cutting edge: Foxp3^+^CD4^+^CD25^+^ regulatory T cells induced by IL-2 and TGF-beta are resistant to Th17 conversion by IL-6. J. Immunol..

[76] Massoud A.H., Charbonnier L.M., Lopez D., Pellegrini M., Phipatanakul W., Chatila T.A. (2016). An asthma-associated IL4R variant exacerbates airway inflammation by promoting conversion of regulatory T cells to TH17-like cells. Nat. Med..

[77] Tarique M., Saini C., Naqvi R.A., Khanna N., Sharma A., Rao D.N. (2017). IL-12 and IL-23 modulate plasticity of FoxP3^+^ regulatory T cells in human Leprosy. Mol. Immunol..

[78] Tang Q., Adams J.Y., Penaranda C., Melli K., Piaggio E., Sgouroudis E., Piccirillo C.A., Salomon B.L., Bluestone J.A. (2008). Central role of defective interleukin-2 production in the triggering of islet autoimmune destruction. Immunity.

[79] Bailey-Bucktrout S.L., Martinez-Llordella M., Zhou X., Anthony B., Rosenthal W., Luche H., Fehling H.J., Bluestone J.A. (2013). Self-antigen-driven activation induces instability of regulatory T cells during an inflammatory autoimmune response. Immunity.

[80] Ye C., Brand D., Zheng S.G. (2018). Targeting IL-2: An unexpected effect in treating immunological diseases. Signal. Transduct Target. Ther..

[81] Chambers E.S., Suwannasaen D., Mann E.H., Urry Z., Richards D.F., Lertmemongkolchai G., Hawrylowicz C.M. (2014). 1alpha,25-dihydroxyvitamin D3 in combination with transforming growth factor-beta increases the frequency of Foxp3^+^ regulatory T cells through preferential expansion and usage of interleukin-2. Immunology.

[82] Urry Z., Chambers E.S., Xystrakis E., Dimeloe S., Richards D.F., Gabrysova L., Christensen J., Gupta A., Saglani S., Bush A. (2012). The role of 1alpha, 25-dihydroxyvitamin D3 and cytokines in the promotion of distinct Foxp3^+^ and IL-10^+^ CD4^+^ T cells. Eur. J. Immunol..

[83] Lu L., Lan Q., Li Z., Zhou X., Gu J., Li Q., Wang J., Chen M., Liu Y., Shen Y. (2014). Critical role of all-trans retinoic acid in stabilizing human natural regulatory T cells under inflammatory conditions. Proc. Natl. Acad. Sci. USA.

[84] Puccetti P., Fallarino F. (2008). Generation of T cell regulatory activity by plasmacytoid dendritic cells and tryptophan catabolism. Blood Cells Mol. Dis..

[85] Schenk U., Frascoli M., Proietti M., Geffers R., Traggiai E., Buer J., Ricordi C., Westendorf A.M., Grassi F. (2011). ATP inhibits the generation and function of regulatory T cells through the activation of purinergic P2X receptors. Sci. Signal..

[86] Grassi F. (2020). The P2X7 Receptor as Regulator of T Cell Development and Function. Front. Immunol..

[87] DeBerardinis R.J., Thompson C.B. (2012). Cellular metabolism and disease: What do metabolic outliers teach us?. Cell.

[88] Michalek R.D., Gerriets V.A., Jacobs S.R., Macintyre A.N., MacIver N.J., Mason E.F., Sullivan S.A., Nichols A.G., Rathmell J.C. (2011). Cutting edge: Distinct glycolytic and lipid oxidative metabolic programs are essential for effector and regulatory CD4^+^ T cell subsets. J. Immunol..

[89] Angelin A., Gil-de-Gomez L., Dahiya S., Jiao J., Guo L., Levine M.H., Wang Z., Quinn W.J., Kopinski P.K., Wang L. (2017). Foxp3 Reprograms T Cell Metabolism to Function in Low-Glucose, High-Lactate Environments. Cell Metab..

[90] Gerriets V.A., Kishton R.J., Johnson M.O., Cohen S., Siska P.J., Nichols A.G., Warmoes M.O., de Cubas A.A., MacIver N.J., Locasale J.W. (2016). Foxp3 and Toll-like receptor signaling balance Treg cell anabolic metabolism for suppression. Nat. Immunol..

[91] Huynh A., DuPage M., Priyadharshini B., Sage P.T., Quiros J., Borges C.M., Townamchai N., Gerriets V.A., Rathmell J.C., Sharpe A.H. (2015). Control of PI(3) kinase in Treg cells maintains homeostasis and lineage stability. Nat. Immunol..

[92] Wei J., Long L., Yang K., Guy C., Shrestha S., Chen Z., Wu C., Vogel P., Neale G., Green D.R. (2016). Autophagy enforces functional integrity of regulatory T cells by coupling environmental cues and metabolic homeostasis. Nat. Immunol..

[93] Shrestha S., Yang K., Guy C., Vogel P., Neale G., Chi H. (2015). Treg cells require the phosphatase PTEN to restrain TH1 and TFH cell responses. Nat. Immunol..

[94] Weinberg S.E., Singer B.D., Steinert E.M., Martinez C.A., Mehta M.M., Martinez-Reyes I., Gao P., Helmin K.A., Abdala-Valencia H., Sena L.A. (2019). Mitochondrial complex III is essential for suppressive function of regulatory T cells. Nature.

[95] Chapman N.M., Zeng H., Nguyen T.M., Wang Y., Vogel P., Dhungana Y., Liu X., Neale G., Locasale J.W., Chi H. (2018). mTOR coordinates transcriptional programs and mitochondrial metabolism of activated Treg subsets to protect tissue homeostasis. Nat. Commun..

[96] Wu D., Luo Y., Guo W., Niu Q., Xue T., Yang F., Sun X., Chen S., Liu Y., Liu J. (2017). Lkb1 maintains Treg cell lineage identity. Nat. Commun..

[97] Timilshina M., You Z., Lacher S.M., Acharya S., Jiang L., Kang Y., Kim J.A., Chang H.W., Kim K.J., Park B. (2019). Activation of Mevalonate Pathway via LKB1 Is Essential for Stability of Treg Cells. Cell Rep..

[98] Zeng H., Yang K., Cloer C., Neale G., Vogel P., Chi H. (2013). mTORC1 couples immune signals and metabolic programming to establish T(reg)-cell function. Nature.

[99] Stancu C., Sima A. (2001). Statins: Mechanism of action and effects. J. Cell Mol. Med..

[100] Vallion R., Divoux J., Glauzy S., Ronin E., Lombardi Y., Lubrano di Ricco M., Gregoire S., Nemazanyy I., Durand A., Fradin D. (2020). Regulatory T Cell Stability and Migration Are Dependent on mTOR. J. Immunol..

[101] Xu T., Stewart K.M., Wang X., Liu K., Xie M., Ryu J.K., Li K., Ma T., Wang H., Ni L. (2017). Metabolic control of TH17 and induced Treg cell balance by an epigenetic mechanism. Nature.

[102] Klysz D., Tai X., Robert P.A., Craveiro M., Cretenet G., Oburoglu L., Mongellaz C., Floess S., Fritz V., Matias M.I. (2015). Glutamine-dependent alpha-ketoglutarate production regulates the balance between T helper 1 cell and regulatory T cell generation. Sci. Signal..

[103] Kitz A., de Marcken M., Gautron A.S., Mitrovic M., Hafler D.A., Dominguez-Villar M. (2016). AKT isoforms modulate Th1-like Treg generation and function in human autoimmune disease. EMBO Rep..

[104] Kerdiles Y.M., Stone E.L., Beisner D.R., McGargill M.A., Ch’en I.L., Stockmann C., Katayama C.D., Hedrick S.M. (2010). Foxo transcription factors control regulatory T cell development and function. Immunity.

[105] Ouyang W., Beckett O., Ma Q., Paik J.H., DePinho R.A., Li M.O. (2010). Foxo proteins cooperatively control the differentiation of Foxp3^+^ regulatory T cells. Nat. Immunol..

[106] Harada Y., Harada Y., Elly C., Ying G., Paik J.H., DePinho R.A., Liu Y.C. (2010). Transcription factors Foxo3a and Foxo1 couple the E3 ligase Cbl-b to the induction of Foxp3 expression in induced regulatory T cells. J. Exp. Med..

[107] Peng M., Yin N., Chhangawala S., Xu K., Leslie C.S., Li M.O. (2016). Aerobic glycolysis promotes T helper 1 cell differentiation through an epigenetic mechanism. Science.

[108] Lu L.F., Boldin M.P., Chaudhry A., Lin L.L., Taganov K.D., Hanada T., Yoshimura A., Baltimore D., Rudensky A.Y. (2010). Function of miR-146a in controlling Treg cell-mediated regulation of Th1 responses. Cell.

[109] Gaddis D.E., Padgett L.E., Wu R., McSkimming C., Romines V., Taylor A.M., McNamara C.A., Kronenberg M., Crotty S., Thomas M.J. (2018). Apolipoprotein AI prevents regulatory to follicular helper T cell switching during atherosclerosis. Nat. Commun..

[110] Wolf D., Gerhardt T., Winkels H., Anto Michel N., Pramod A.B., Ghosheh Y., Brunel S., Buscher K., Miller J., McArdle S. (2020). Pathogenic Autoimmunity in Atherosclerosis Evolves from Initially Protective ApoB-Reactive CD4^+^ T-Regulatory Cells. Circulation.

[111] Xu J., Yang Y., Qiu G., Lal G., Wu Z., Levy D.E., Ochando J.C., Bromberg J.S., Ding Y. (2009). c-Maf regulates IL-10 expression during Th17 polarization. J. Immunol..

[112] Neumann C., Blume J., Roy U., Teh P.P., Vasanthakumar A., Beller A., Liao Y., Heinrich F., Arenzana T.L., Hackney J.A. (2019). c-Maf-dependent Treg cell control of intestinal TH17 cells and IgA establishes host-microbiota homeostasis. Nat. Immunol..

[113] Mor A., Planer D., Luboshits G., Afek A., Metzger S., Chajek-Shaul T., Keren G., George J. (2007). Role of naturally occurring CD4^+^ CD25^+^ regulatory T cells in experimental atherosclerosis. Arter. Thromb. Vasc. Biol..

[114] Mor A., Luboshits G., Planer D., Keren G., George J. (2006). Altered status of CD4^+^CD25^+^ regulatory T cells in patients with acute coronary syndromes. Eur. Heart J..

[115] Mailer R.K.W., Gistera A., Polyzos K.A., Ketelhuth D.F.J., Hansson G.K. (2017). Hypercholesterolemia Induces Differentiation of Regulatory T Cells in the Liver. Circ. Res..

[116] Parathath S., Mick S.L., Feig J.E., Joaquin V., Grauer L., Habiel D.M., Gassmann M., Gardner L.B., Fisher E.A. (2011). Hypoxia is present in murine atherosclerotic plaques and has multiple adverse effects on macrophage lipid metabolism. Circ. Res..

[117] Dang E.V., Barbi J., Yang H.Y., Jinasena D., Yu H., Zheng Y., Bordman Z., Fu J., Kim Y., Yen H.R. (2011). Control of T(H)17/T(reg) balance by hypoxia-inducible factor 1. Cell.

[118] Lee J.H., Elly C., Park Y., Liu Y.C. (2015). E3 Ubiquitin Ligase VHL Regulates Hypoxia-Inducible Factor-1alpha to Maintain Regulatory T Cell Stability and Suppressive Capacity. Immunity.

[119] Salminen A., Kauppinen A., Kaarniranta K. (2015). 2-Oxoglutarate-dependent dioxygenases are sensors of energy metabolism, oxygen availability, and iron homeostasis: Potential role in the regulation of aging process. Cell Mol. Life Sci..

[120] Kimura T., Kobiyama K., Winkels H., Tse K., Miller J., Vassallo M., Wolf D., Ryden C., Orecchioni M., Dileepan T. (2018). Regulatory CD4^+^ T Cells Recognize MHC-II-Restricted Peptide Epitopes of Apolipoprotein B. Circulation.

[121] Kimura T., Tse K., McArdle S., Gerhardt T., Miller J., Mikulski Z., Sidney J., Sette A., Wolf D., Ley K. (2017). Atheroprotective vaccination with MHC-II-restricted ApoB peptides induces peritoneal IL-10-producing CD4 T cells. Am. J. Physiol. Heart Circ. Physiol..

